# Ligands of the Neuropeptide Y Y2 Receptors as a Potential Multitarget Therapeutic Approach for the Protection of the Neurovascular Unit Against Acute Ischemia/Reperfusion: View from the Perspective of the Laboratory Bench

**DOI:** 10.1007/s12975-021-00930-4

**Published:** 2021-07-22

**Authors:** Łukasz Przykaza, Ewa Kozniewska

**Affiliations:** Laboratory of Experimental and Clinical Neurosurgery, Mossakowski Medical Research Institute Polish Academy of Sciences, A. Pawińskiego Str. 5, 02-106, Warsaw, Poland

**Keywords:** Cerebral ischemia/reperfusion, Neuropeptide Y Y2 receptors, Neuroprotection, Neurovascular unit, Stroke therapy

## Abstract

Ischemic stroke is the third leading cause of death and disability worldwide, with no available satisfactory prevention or treatment approach. The current treatment is limited to the use of “reperfusion methods,” i.e., an intravenous or intra-arterial infusion of a fibrinolytic agent, mechanical removal of the clot by thrombectomy, or a combination of both methods. It should be stressed, however, that only approximately 5% of all acute strokes are eligible for fibrinolytic treatment and fewer than 10% for thrombectomy. Despite the tremendous progress in understanding of the pathomechanisms of cerebral ischemia, the promising results of basic research on neuroprotection are not currently transferable to human stroke. A possible explanation for this failure is that experiments on in vivo animal models involve healthy young animals, and the experimental protocols seldom consider the importance of protecting the whole neurovascular unit (NVU), which ensures intracranial homeostasis and is seriously damaged by ischemia/reperfusion. One of the endogenous protective systems activated during ischemia and in neurodegenerative diseases is represented by neuropeptide Y (NPY). It has been demonstrated that activation of NPY Y2 receptors (Y2R) by a specific ligand decreases the volume of the postischemic infarction and improves performance in functional tests of rats with arterial hypertension subjected to middle cerebral artery occlusion/reperfusion. This functional improvement suggests the protection of the NVU. In this review, we focus on NPY and discuss the potential, multidirectional protective effects of Y2R agonists against acute focal ischemia/reperfusion injury, with special reference to the NVU.

## Introduction

Ischemic stroke is a major clinical and socioeconomic problem of the aging population in industrialized countries. The World Health Organization (WHO) estimates that every year approximately 15 million people across the world suffer from ischemic stroke, 5 million of whom die, which makes ischemic stroke the third leading cause of death worldwide [1, 2]. Many of those who survive an ictus become incapacitated, facing difficulties in performing daily activities.

The current treatment for ischemic stroke is limited to the use of “reperfusion methods”, i.e., an intravenous or intra-arterial infusion of a thrombolytic agent such as a recombinant tissue plasminogen activator (rt-PA, alteplase); mechanical removal of the clot by surgical intravascular intervention; or a combination of these methods [2–4]. These therapies involve a serious risk of complications, such as hemorrhagic transformation and secondary brain edema, which may further exacerbate a patient’s condition or lead to death [5]. In addition, it should be stressed that only approximately 5% of all acute strokes are eligible for fibrinolytic treatment [6] and fewer than 10% for thrombectomy [7].

Despite the tremendous progress in understanding the pathomechanisms of cerebral ischemia, the promising results of basic research on neuroprotection are not currently transferable to human stroke. One explanation for this failure is that experiments on in vivo animal models involve healthy young animals that do not suffer from arterial hypertension, hyperlipidemia, diabetes, or cardiac arrhythmia, all of which are well-known risk factors of human stroke [8]. This, in addition to the single-target treatment approach, without protection of glial cells and blood vessels, might be the major reason for the failure of experimentally recognized neuroprotective strategies to be transferred to the bedside.

It has become obvious that basic research should take into consideration the complexity of the ischemic pathophysiology and its accompanying diseases. Ischemia-related processes affect all cell types in the brain, including the immune system [9, 10]. This fact has been overlooked for a long time as most experimental studies on protecting against ischemia only focused on the protection of neurons [11]. This attitude changed when the concept of the multicomponent neurovascular unit (NVU) was introduced in 2001 to accentuate the importance of the strong and unique coupling between brain cells (neurons and astrocytes) and the cerebral microvasculature (https://www.ninds.nih.gov/About-NINDS/Strategic-Plans-Evaluations/Strategic-Plans/Stroke-Progress-Review-Group). However, to date, no therapy targeting the NVU has been proposed for patients with acute stroke.

Over the last few decades, attention has been paid to therapies based on endogenous protective and repair processes [12]. These processes, occurring in parallel with a damaging cascade of excitotoxicity, involve the release of the inhibitory neurotransmitter GABA and adenosine and the activation of ATP-sensitive K^+^ channels to oppose excitotoxicity, anti-inflammatory and anti-apoptotic signaling, and repair/regeneration processes.

One class of endogenous substances with potential protective properties are neuropeptides, which serve as cotransmitters or neuromodulators under physiological circumstances and are known to also be activated in response to brain injury and in neurodegenerative diseases [13]. Due to the absence of specific reuptake mechanisms and their special kinetics, neuropeptides exert long-lasting effects. One of the most abundant neuropeptides in the brain, expressed by multiple neuronal systems, is neuropeptide Y (NPY) [14, 15]. The biological actions of this peptide in mammals are mediated by 5 types of specific receptors, namely Y1, Y2, Y4, Y5, and Y6 [16]. It has been demonstrated that the activation of Y2 receptors (Y2R) by a specific ligand decreases the volume of the postischemic infarction and improves gait parameters in rats with arterial hypertension subjected to middle cerebral artery occlusion/reperfusion [17, 18]. The functional improvement suggests the protection of the NVU. In this minireview, we focus on Y2R and discuss the potential, multidirectional protective effects of the specific ligands of these receptors against acute focal ischemia/reperfusion injury, with special reference to the NVU.

### On How the Ischemic Cascade Affects the Neurovascular Unit

The NVU is a structural and functional entity composed of neurons, astrocytes, and the microvascular endothelium, which, together with perivascular astrocytic foot processes, pericytes, and the extracellular matrix, form the blood–brain barrier (BBB). These structural components are intimately and reciprocally linked to each other to ensure brain homeostasis, including an efficient system of microflow control [19]. Disintegration and dysfunction of the NVU due to cerebral ischemia leads to loss of the normal function of the ischemic region. During the first minutes of focal cerebral ischemia, the ischemic region becomes spatially divided into an ischemic core with insufficient blood flow (oxygen and glucose supplies) to retain cellular integrity and a surrounding penumbra where the collateral flow maintains sufficient oxygen and glucose levels to ensure cellular integrity for some time. The cells located in the ischemic core cannot be salvaged and undergo necrosis, while those in the penumbra die slowly over time via apoptosis and may be rescued. Therefore, the aim of therapy in stroke is to save the penumbra. The ischemia-induced processes occurring in the core of the infarct include the failure of ATP production and, in consequence, the inability to maintain the membrane potential, leading to Na^+^ and Ca^2+^accumulation within cells and an increase of the extracellular K^+^ concentration.

Intracellular accumulation of ions is associated with the passive influx of water into cells, i.e., cytotoxic/ionic edema and disintegration of neurovascular unit [20]. Edema can result in the compression of microvessels in the penumbra in the vicinity of the core of the infarct and can further decrease perfusion. Edema can also decompensate the intracranial volume-pressure relationship, resulting in increases of intracranial pressure and secondary vascular compression. The increase of the intracellular concentration of Ca^2+^ results in an excessive release of the excitatory aminoacid glutamate, the generation of oxygen and nitrogen-free radicals, and the activation of Ca^2+^-dependent catabolic enzymes. In addition, glial cells deprived of ATP lose their ability to buffer potassium ions and to remove glutamate from the extracellular space [19]. The glutamate accumulating and diffusing in the extracellular space leads to excitotoxicity, whereas the increased concentration of extracellular potassium ions together with glutamate triggers peri-infarct depolarizations (PIDs) [21].

Both glutamate and PIDs are the main factors that gradually recruit the penumbral zone to the ischemic core. Glutamate, diffusing in the interstitial space, reaches the penumbra and excites ionotropic and metabotropic receptors in a non-physiological, excessive, and prolonged manner, resulting in energy depletion, in an influx of Ca^2+^ into the cytosol, and subsequently, the gradual death of cells located in the penumbra due to the activation of Ca^2+^-dependent catabolic enzymes and the induction of apoptosis [22, 23].

Intracellular accumulation of Ca^2+^ occurs not only as a result of prolonged activation of NMDA receptors. There are at least three other Ca^2+^ permeable membrane channels activated during brain ischemia. One of them is acid-sensing ion channel (ASIC1a), activated due to metabolic acidosis, largely caused by recurrent episodes of PIDs [24, 25]. Opening of this channel significantly increases the intracellular pool of calcium ions, and contributes to neuronal death in the penumbra [26, 27]. The remaining two channels belong to the superfamily of cation/Ca^2+^ exchangers, represented by K^+^-independent (NCXs) and K^+^-dependent Na^+^-Ca^2+^ exchangers (NCKXs) [28, 29]. Both channels are bi-directional membrane ion transporters. Depending on the energetic state of the cell and ionic intra- and extracellular fluid composition, these channels may conduct in forward (calcium exit) or reverse (calcium entry) mode. The participation of these channels in ischemic neuronal death has been well documented [30–32].

The loss of energy/ionic homeostasis disrupts the communication of neurons with glial and endothelial cells. Astrocytes exposed to glutamate toxicity, similar to neurons, suffer from Ca^2+^overload, cytotoxic edema, and mitochondrial depolarization, followed by free radical damage [33]. In addition, the cerebral microvasculature in the penumbra loses its physiological regulation due to the impairment of endothelial/smooth muscle cells function caused by oxidative/nitrosative stress, amplified by progressive inflammatory processes [34–36]. In consequence, excitotoxicity causes the disintegration and dysfunction of the NVU.

Transient PIDs, spreading from the core into the penumbra, also promote the enlargement of the core, by recruiting penumbra. It has been demonstrated that the infarction volume is correlated positively with the number of PIDs [37, 38]. PIDs also result in damage to the NVU due to the activation of metalloproteinases and disruption of the BBB [39]. The severity of ischemic BBB damage and the degree of vasogenic edema have been reported to correlate with the number of PID episodes [39]. PIDs may also deepen microflow deficits in the cerebral cortex in the penumbra due to the constriction of microvessels [40, 41]. The degradation of the extracellular matrix of microcirculatory endothelial cells by metalloproteinases and the decreased synthesis of integrin α_6_β_4_ in astrocytes disrupt astrocyte-endothelium communication [42].

The damage to the NVU in the penumbra can be exacerbated by reperfusion [43–45]. This phenomenon, known as reperfusion injury, has a multifactorial etiology. First, during reperfusion, complex PIDs and cycles of hyperemia/hypoperfusion, with elevations of extracellular potassium ions to vasoconstrictive concentrations, continue and deepen the neurovascular uncoupling [41]. Reperfusion and PIDs may also lead to further dysfunction of the vascular inwardly rectifying potassium (Kir) channels, which adds to the deterioration of the neurovascular coupling [46–48].

In addition, leukocytes activated by oxidative stress upon the restoration of tissue perfusion play a critical role in reperfusion injury. Leukocytes release metalloproteinases, which, in addition to having neurotoxic properties, degrade the extracellular matrix and tight junctions proteins of the BBB [49]. The leaky BBB allows more leukocytes to infiltrate brain tissue, where they release proinflammatory cytokines and cause inflammation [50]. Furthermore, activated leukocytes express adhesion molecules and interact with aggregating platelets, forming leukocyte-platelet aggregates that adhere to the inner walls of the capillaries and venules, resulting in an obstruction of the microcirculation [35, 51]. Additionally, activated platelets may release vasoconstrictors, such as ATP and thromboxane A_2_, and chemotactic mediators, attracting migrating leukocytes [52, 53]. Due to the no-reflow phenomenon, the microvessels will be not perfused despite the successful recanalization of the clotted larger inflow vessel. No-reflow may also be caused by compression of the smallest microvessels by the swollen perivascular astrocytic end-feet processes.

Activated leukocytes produce oxygen-free radicals (mainly superoxide anion, O^2−^). The main O^2−^-producing enzyme found in leukocytes and macrophages is NADPH oxidase, which also occurs abundantly in endothelial cells stimulated by cytokines and in the presence of an increased intracellular Ca^2+^ concentration [54–56]. Superoxide anion interacts with nitric oxide to produce the highly toxic nitrogen-free radical peroxynitrite [57–59].

All of these processes can lead to hemorrhagic transformation during reperfusion and contribute to the aggravation of BBB damage, vasogenic brain edema, and increase of the influx of leukocytes into the brain parenchyma. These complications significantly reduce the usage of rt-PA [45].

### Basic Concepts of a Multitarget Treatment of Stroke to Ensure NVU Protection

**Fig. 1:**
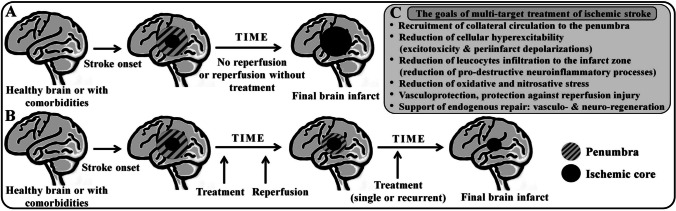
The time course of spatio-temporal infarct evolution without (A) and with a protective treatment (B) and fundamental concepts of the ischemic stroke therapy (C). The detailed explanation is provided in the text

Taking into consideration the mechanisms responsible for the ischemic brain damage and the endogenous processes that counteract the evolution of ischemia-related degeneration, we attempt to characterize the ideal protective compound (Fig. 1). The protectant should be applied in the early emergency phase, when the viable penumbra still exists, to support the recruitment of collaterals to prolong the viability of the penumbra. At the same time, the protectant should inhibit the overstimulation of neurons and activation of glial cells by counteracting the cascade associated with the Ca^2+^ overload of these cells. Stabilization of the membrane potentials and ionic/water homeostasis of cells located in the penumbral/ischemic area are the most important goals in this early phase. The substance should also reduce oxidative/nitrosative stress and exhibit anti-inflammatory properties. Deactivation of leukocytes will significantly reduce the level of free radical stress in inflammatory cells and in the endothelium by reducing the concentration of proinflammatory cytokines. All of these actions will stabilize the integrity of the NVU during the acute phase of ischemic stroke. The ideal protective compound should also inhibit the adverse phenomena associated with reperfusion, such as the propagation of PIDs and activation of proinflammatory leukocytes, and, in the later phase of reperfusion, support endogenous repair processes, such as angiogenesis and neurogenesis. Selective agonists of Y2R seem to fulfill most of these criteria, as will be discussed below.

## The Protective Potential of Y2 Receptors Against Acute Ischemic NVU Injury

### The Distribution and Physiological Functions of Y2 Receptors in the Central Nervous System (CNS)

Neuropeptide Y Y2 receptor is a member of the class A family of seven-transmembrane G protein–coupled receptors. The human Y2R consists of 381 amino acids, and its preferred native agonists (unselective) are NPY and peptide YY. Interestingly, the endothelial serine protease dipeptidyl peptidase 4 cleaves full-length NPY1-36 into NPY3-36, which selectively activates Y2 receptors [60, 61]. There are also several synthetic Y2R-specific ligands, such as the C-terminal NPY fragment (13–36) [NPY13-36] and other peptide and nonpeptide compounds [62, 63]. The genes that encode Y2R are localized in the 4q31 chromosomal segment in humans, and in rat and mouse, the Y2R gene is in the 2q31 and 3 E3 segments, respectively [64–67]. More than 92% of the amino acid sequence of Y2R is similar across mammals, which emphasizes the importance of Y2R in critical metabolic processes [68].

The distribution and density of the Y2 receptor in the CNS varies depending on the anatomical location. In humans, in situ hybridization studies of postmortem brain tissues have revealed high levels of the Y2R-mRNA signal in neurons throughout the cortical regions, the CA2 region of the hippocampus, the lateral geniculate nucleus, the amygdala, the substantia nigra, the hypothalamus, and the cerebellum and low levels of the Y2R-mRNA in the striatum [69]. Similarly, in the rat brain, Y2R is widely expressed. Y2R-mRNA is located within the hippocampus, hypothalamus, and amygdala. In the cerebral cortex, the signal is present at a low level [70]. Data obtained from agonist-induced binding autoradiography of Y2R in the rat brain further supports the above mentioned distribution based on transcript identification. Functional detection of Y2R confirms the distribution of the receptor throughout the cortical and subcortical parts of the rat brain [71]. In mice, immunohistochemical studies of the location of Y2R also showed the common presence of this receptor in many brain regions, including cortical areas, the basal forebrain, the nucleus accumbens, the amygdala, the hippocampus, the hypothalamus, and the substantia nigra compacta. At the synaptic level, the Y2R protein can be found both pre- and postsynaptically, with the predominance of presynaptic location [72, 73].

Y2R has been also detected in human cerebral astrocytes and in the neurons of monkey and rat spinal cords [74, 75]. Y2 receptors are also present in the endothelium of cerebral blood vessels, as demonstrated by functional study on isolated rat middle cerebral arteries (MCA), in which a selective agonist of Y2R was used [76]. According to this study, Y2R activation leads to a dose-dependent vasodilation of rat MCAs. This response is mediated by endothelial nitric oxide (NO) and the activation of cGMP-dependent relaxation of smooth muscle cells.

Y2R is involved in the modulation of many physiological processes in the CNS, such as stress and emotional reactions, circadian rhythms, memory processes, energetics/appetite, and blood pressure regulation [77, 78]. The main function of Y2R in all these processes is the inhibition of the release of neurotransmitters from presynaptic terminals, among others, the inhibition of the release of excitatory glutamic acid [79, 80]. This observation is the basis for research on the neuroprotective potential of neuropeptide Y and its analogs that selectively activate Y2R.

### Y2 Receptor Stimulation Exerts Protective Effects in Experimental Stroke Models

**Fig. 2:**
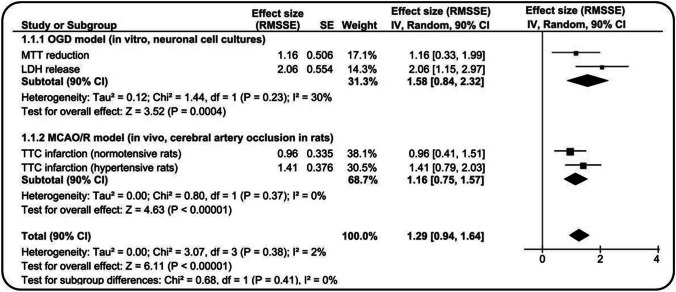
The forest plot presenting the comparison of the effects of a selective agonist of Y2 receptor – NPY 13–36 on the ischemic damage in vitro (OGD, neuronal cell culture) measured as cellular mortality-survival level and in vivo (MCAO/R in two strains of rats) measured as infarction volume at 72 h after 90-min focal cerebral ischemia/reperfusion. The results of each model have been calculated to a common measure, a standardized effect size – RMSSE. On the forest plot, squares and horizontal lines represent individual study RMSSE and 90% confidence intervals (CI) and diamonds represent overall weighted mean RMSSE and 90% CI. The random-effects subgroup meta-analysis (in vitro subgroup and in vivo subgroup) revealed high significances of overall weighted mean effect sizes (*P* = 0.00001–0.0004) and absence of significant heterogeneity (*I*^2^) between them (total) and within the subgroups/models (subtotal). Note that all confidence intervals are overlapping and Total *I*^2^ = 2%, *P* = 0.38. This result demonstrates comparable effects magnitude of treatment with Y2R agonist both in models consisting only of neurons (neuronal cultures) and in models comprising whole neurovascular unit (rats). There is also no difference between normotensive and hypertensive rats. This suggests that Y2R activation protects not only neurons but also other components of NVU against ischemic damage, also in the rat model of essential arterial hypertension. The comparatively analyzed Y2R treatment models have parallel types and numbers of groups (“only ischemia,” “treatment during ischemia,” and “treatment 30 min after ischemic episode”). NPY13-36 doses applied: OGD model – 1 µM added to the culture media; MCAO/R models – 10 µg/6 µl administered into the lateral cerebral ventricle. RMSSE – the root mean square standardized effect; SE – the standard error of effect size; CI – confidence interval; heterogeneity was tested by Cochrane’s Q (chi^2^) at a significance level of *P* < 0.09 and quantified by *I*^2^ – inconsistency value, which tells what part of the observed variance reflects the true variation of effects, not caused by sampling error (*I*^2^ ≥ 25% is a considerable heterogeneity) [Higgins JPT, et al., 2003]; OGD – 3-h oxygen–glucose deprivation of mouse primary neuronal cortical cultures model; MTT – 3-[4,5-dimethyl-thiazol-2-yl]-2,5-diphenyltetrazolium bromide, a degree of its reduction is an indicator of cell culture viability; LDH – lactate dehydrogenase, a concentration of which in the culture media indicates the level of cells damage. MCAO/R – 90-min middle cerebral artery occlusion with reperfusion model in rats; TTC – 2,3,5-triphenyltetrazolium chloride, TTC staining indicates the extent of infarct volume in brain coronal sections. Domin H., et al. (2017) (OGD and MCAO/R in normotensive rats models); Przykaza L., et al. (2016) (MCAO/R in hypertensive rats model). The statistical procedure of the subgroup mini meta-analysis [Goh J.X. et al., 2016] by generic inverse variance method was conducted as follows: three groups were considered for analysis, based on creation of three-group one-way ANOVA parallel models. In the OGD model: OGD group (control group), Y2R agonist before and after OGD, Y2R agonist 30 min after OGD; in both MCAO/R models: MCAO/R group (control group), MCAO/R Y2R agonist during ischemia, MCAO/R Y2R agonist 30 min after reperfusion. Data from the results of the OGD model (means and standard errors of the means) were extracted morphometrically from the published graphs using the WebPlotDigitizer off-line software [Drevon D., et al., 2017]. Group sizes were taken from the published text (in MCAO/R hypertensive rats model the size of groups is: *n* = 10, *n* = 7, *n* = 6). In each one-way ANOVA model, the post hoc analysis revealed significant intergroup diversity between control and both groups with Y2R agonist (same effects directions). Based on means, SEMs and outputs of one-way ANOVA models and standardized effect sizes were calculated for each entire ANOVA model – the root mean square standardized effect (RMSSE). Standard errors of effect sizes were estimated based on modification of Wald test: SE = effect size/*Z*, where *Z* is the standard normal deviate corresponding to the *P* value (one-tailed normal distribution table) given from one-way ANOVA F-test [Higgins J.P.T., et al., 2019]. The subgroups were assigned based on the in vitro or in vivo type of model experimental procedure. Subgroup random-effects meta-analysis (with the related forest plot) was done using the RevMan 5.3 software (Review Manager 5.3, Copenhagen: The Nordic Cochrane Centre, The Cochrane Collaboration, 2014)

Experimental research provides evidence that the agonists of Y2Rs may be promising candidates for stroke treatment and may include multiple targets [17, 18, 81]. There is a direct demonstration of the protective effect of Y2R stimulation against in vitro and in vivo excitotoxicity and in cerebral ischemia studies, in which anY2R selective agonist (NPY13-36) was used [17, 81]. These studies demonstrated for the first time that NPY13-36 could exert significant protection on neurochemical, structural, and behavioral changes after stroke. The protective effects were observed when the compound was applied during ischemia and in the reoxygenation/reperfusion phase. The similar result was found in animals with essential arterial hypertension [18]. Interestingly, a significant increase in NPY immunostaining has been reported in the cerebral cortex in peri-infarct regions after permanent middle cerebral artery occlusion in rats [82]. Figure 2 presents an analysis of the results of the degree/extent of the damage in in vitro and in vivo models of cerebral ischemia. A subgroup mini meta-analysis (two subgroups, both with *n* = 2 studies) was performed according to Higgins JPT et al. [83, 84] and Goh JX et al. [85]. This analysis revealed significantly large effect sizes (total effect size: RMSSE = 1.29, *Z* = 6.11, *P* < 0.00001) and a high resemblance of the efficiency magnitude of the action of NPY13-36 against ischemic cell death in all the applied modes of the treatment (total heterogeneity: *I*^2^ = 2%, *P* = 0.38). This result demonstrates the comparable effects of the magnitude of treatment with the Y2R agonist in models consisting only of neurons (neuronal cultures) and in models including the whole neurovascular unit (rats). There was also no difference between normotensive and hypertensive rats (subtotal heterogeneity within the MCAO/R subgroup: *I*^2^ = 0%, *P* = 0.37). This finding suggests that the Y2R agonist protects not only neurons but also other components of the NVU against ischemia/reperfusion, and it is only as effective in protecting the ischemic brain of the animals with concomitant arterial hypertension as that without comorbidities. Despite the lack of significant total heterogeneity, NPY13-36 was probably more coherently effective in in vivo models, which better explains its total effect—as indicated by the large statistical weight of the in vivo subgroup (subtotal weight = 68.7%)—in comparison with the in vitro subgroup (subtotal weight = 31.3%).

### Putative Mechanisms of the Y2R-Related Protective Effects in Experimental Stroke Models

#### Recruitment of Collateral Flow to the Penumbra by the Activation of Endothelial NOS

The penumbra, which is supplied with some oxygen during ischemia, suffers from more oxidative/nitrosative stress than the core, which results in a reduction of endothelial nitric oxide production [86, 87] and the constriction of the blood vessels supplying the penumbra. Stimulation of Y2R by a selective agonist might increase collateral flow to the penumbra by increasing endothelial NO production and vasodilation [76]. The effect of the selective stimulation of Y2R on collateral flow in the ischemic brain has not been reported to date. However, based on the temporal profile data regarding the functional state of endothelial NO synthase after MCA occlusion/reperfusion in rat, the improvement of collateral flow after stimulation of Y2R should be possible for at least 6 h after ischemia/reperfusion. Yagita et al. have shown that endothelial NO synthase (eNOS) mRNA and protein expression, as well as eNOS-ser117 phosphorylation, increases by a few-fold in the ischemic penumbra up to 6 h after MCA occlusion/reperfusion [88]. NO released after Y2R stimulation, will not only increase blood flow to the penumbra but also will inhibit platelet and leukocytes adhesion and scavenge oxygen free radicals [89]. This, in turn, will oppose vessel plugging and no-reflow of the microcirculation. All of these actions should improve the survival of the penumbra.

#### Counteraction of the Overexcitation of Cells in the Penumbra by Inhibiting cAMP/PKA Activity

Y2R is a metabotropic receptor associated with G_iα_ subunit of the G protein. The activation of this receptor results in inhibition of the activity of adenylyl cyclase (AC), and decrease in the intracellular cyclic adenosine monophosphate (cAMP) concentration and the associated protein kinase A (PKA) activity. This ultimately decreases neuronal excitation. As already mentioned, neuronal overexcitation during the course of ischemia is mainly caused by glutamic acid, which stimulates the NMDA, AMPA, and metabotropic receptors, and by unbuffered K^+^, which accumulates in the extracellular space and increases the spread of PIDs. Although the main intracellular mediator associated with the stimulation of NMDA receptors is Ca^2+^, the intracellular concentration of cAMP also increases when these receptors are activated. The increase in intracellular concentration of cAMP occurs secondary to the stimulation of adenylyl cyclase by the Ca^2+^/calmodulin complex [90].

Cyclic AMP has been demonstrated to be an important intracellular messenger of the signaling associated with the activation of NMDA receptors. It has been shown that intracellular cAMP enhances the release of excitatory neurotransmitters by increasing Ca^2+^ secretion from the smooth endoplasmic reticulum (SER) as a result of the phosphorylation of ryanodine receptors (RyR) by PKA and the increased opening probability of this channel [91]. In addition, activation of the adenylyl cyclase/cAMP/PKA pathway facilitates exocytosis [92]. Moreover, cyclic AMP and protein kinase A enhance transmission through the NMDA receptor channel, which indicates that the excitability of this channel is increased by PKA-dependent phosphorylation [93]. Similarly, activation of AC/cAMP signaling in striatal neurons facilitates corticostriatal transmission and potentiates the excitatory effects of activation of the NMDA and AMPA receptors [94]. It is widely known that neurons located in the striatum synthesize large amounts of adenylyl cyclase and that an increase in the intracellular cAMP level increases their excitation [95, 96]. Importantly, increases in AC activity and in the intracellular cAMP concentration have also been observed in cerebral ischemia models [97]. This cellular excitation can also be potentiated by inhibiting the conductance of Kir channels through activation of cAMP/PKA pathway [98]. An increase in the intracellular cAMP level was also detected during the course of cortical spreading depression caused by KCl application on the rat cerebral cortex surface in vitro and in vivo [99, 100] and during the course of epileptic-like audiogenic seizures in epilepsy-prone rats [101]. On the other hand, the acute increase of the intracellular cAMP concentration in nonexcitable astrocytes will attenuate glutamate reuptake through endocytosis of glutamate transporters GLT-1 and GLAST [102–104]. This enhances and prolongs the excitation of the postsynaptic neuron.

In addition, acute increase of the activity of astrocytic cAMP/PKA signaling leads to phosphorylation of aquaporin-4 (AQP4), which ultimately increases water permeability of the membrane, and promotes cytotoxic edema of astrocytes [20, 105, 106]. In regard to astrocytic AQP4, it has been demonstrated that this protein significantly contributes to the propagation of depolarizing waves by increasing the extracellular concentration of K^+^ [107].

Taken together, administration of cAMP inhibitors (such as an agonist of Y2R) during acute phase of cerebral ischemia, should suppress ischemia-induced excitotoxicity and cytotoxic edema.

There are no data available in the accessible literature on the inhibition of postischemic excitotoxicity and/or PIDs after stimulation of Y2R. However, Y2R stimulation has been demonstrated to suppress excitotoxicity in epileptic attacks, both in experiments as well as in patients suffering from epilepsy [108–112]. The intracellular mechanisms connected with the suppression of neuronal excitability during the stimulation of Y2 receptors consist, in addition to the direct inhibition of cAMP/PKA signaling, of attenuation of the conductance of the N and P/Q calcium channels [78, 80] and an increase in the conductance of the Kir channels [113]. Inhibition of calcium channels results in decreased exocytosis, whereas increased conductance of the Kir channels leads to hyperpolarization of the neuronal membrane and decreased excitability of the cell. Moreover, decrease of the cAMP/PKA signaling may also suppress the conductance of ASIC1a channels and diminish toxic calcium overload [114, 115]. Reductions of ischemic energetic/ionic disturbances and excitotoxicity, achieved through Y2R activation, should protect the cross-talk between the neuron and the astrocyte as the most important part of the neurovascular unit coherence.

#### Inhibition of Oxidative/Nitrosative Stress, Neuroinflammation, and Apoptosis

Oxidative and nitrosative stress during ischemia are consequences of excitotoxicity and an increased intracellular Ca^2+^ concentration, which stimulate cyclooxygenase and neuronal nitric oxide synthase (nNOS) to generate prostanoids and NO, respectively [86, 87]. During the generation of these compounds, a free radical superoxide is formed. The reaction of the superoxide anion with NO results in the production of a very aggressive nitrogen-free radical peroxinitrate. Peroxynitrite promotes lipid peroxidation, mitochondrial and DNA damage, protein nitration and oxidation, depletion of antioxidant reserves, and breakdown of the blood–brain barrier [59]. These free radicals are produced during ischemia, mainly in the penumbra and particularly upon reperfusion [86]. Although there is no direct proof that stimulation of Y2R receptors results in the inhibition of lipid peroxidation during ischemia, such an effect has been demonstrated in the hippocampus and in the prefrontal cortex in mice after an intraventricular injection of amyloid-β [116].

Reactive oxygen/nitrogen species are known to activate sterile inflammation, which means activation of resident microglia and infiltration of peripheral leukocytes into the brain parenchyma [117]. In this context, it has been reported that peripheral monocytes express Y2 receptors and that the stimulation of these receptors can significantly reduce the recruitment of monocytes into the brain [118]. According to recently published data, selective activation of Y2 receptors decreased the number of activated microglia and inducible NOS positive cells, as well as reduced the levels of proinflammatory TNF-alpha and NF-kB in the brain in a mouse model of Huntington’s disease [119]. All in all, these data strongly suggest that activation of Y2R in the acute stroke may inhibit neuroinflammation.

Furthermore, inhibition of calpain/caspase 3 apoptotic pathway observed after administration of a selective Y2R agonist in in vitro model of brain ischemia [17, 61] points to a direct inhibition of apoptosis, the main mechanism of cell death in the penumbra. In the context of apoptosis, it is worth to mention that selective stimulation of Y2R is protective against methamphetamine neurotoxicity, up- and downregulating the protein levels of anti-apoptotic Bcl-2 and pro-apoptotic Bax, respectively [120].

#### Stimulation of the Endogenous Repair Processes: Angiogenesis and Neurogenesis

Angiogenesis is a complex process involving the formation of new blood vessels and is regulated by many growth factors. The most important of these growth factors are basic fibroblast growth factor (bFGF) and vascular endothelium growth factor (VEGF). In the adult organism, angiogenesis is stimulated mainly under pathological situations.

Postischemic damage to the BBB induces regenerative angiogenesis in the mature rat brain [121, 122] as well as in stroke patients, as documented by histological and immunohistochemical studies of postmortem tissue [123]. Experimental studies on animal models show that angiogenesis can be induced as soon as the first day after an ischemic episode [121, 122] and is postulated to be one of the endogenous regenerative processes after experimental and human stroke [124, 125].

Y2R agonists are known to play a role in the induction/progression of angiogenesis [125–128]. The potency and efficacy of Y2R ligands in stimulating angiogenesis have been shown to be similar to FGF and VEGF [126, 129, 130]. It has been suggested that NPY Y2R ligands may act upstream of VEGF and FGF and may be a key factor or master “on switch” to initiate angiogenesis [126, 131]. Stimulation of Y2R has been shown to induce angiogenesis through endothelial cell proliferation, survival, and migration [126, 132, 133]. There are no data on the stimulation of postischemic angiogenesis by agonists of Y2R in the brain, but the positive effects of Y2R stimulation on postischemic angiogenesis in the myocardium and skeletal muscles are well documented [61, 126, 128]. Furthermore, Robich MP et al. proved a significant decrease of antiangiogenic factors endo- and angiostatin—in the ischemic myocardium of the animals treated with selective agonist of Y2R [61]. Thus, it can be assumed with high probability that activation of Y2R will result in the stimulation of angiogenesis in the penumbra in ischemic/postischemic brain.

As far as adult neurogenesis is concerned, there is a good evidence that NPY stimulates neurogenesis and this process depends mainly on the activation of Y1 receptor, but some, although scanty, publications indicate that Y2 receptors may be also involved [134, 135]. Alvaro et al.’s study has shown that retinal neural cell proliferation increased twofold when treated with a selective Y2R agonist NPY13-36 and this effect was completely abolished by Y2R antagonist [135]. As similar, but less potent effect was caused by administration of Y1 and Y5 receptor agonists, the authors concluded that NPY stimulated neurogenesis through an oligomer composed by Y1, Y2, and Y5 receptors. In the case of an oligomer receptor, administration of an antagonist of one of the components of the oligomer is enough to block this complex. The possibility that NPY receptors form homo- or heterodimers was suggested by some functional and molecular studies [133, 136].

In the context of angio- and neurogenesis, it should be mentioned that these processes are related. It has been demonstrated that cerebral endothelial cells activated by ischemia promote proliferation and differentiation of neural stem cells, while neural progenitor cells isolated from the ischemic subventricular zone promote angiogenesis [137].

Although there are no data on the direct effect of Y2R stimulation on postischemic neurogenesis in the brain, it has been documented that selective Y2R agonist upregulated expression of brain-derived neurotropic factor (BDNF), one of the most important stimulators of neurogenesis in the adult brain, in the mouse model of Huntington’s disease [119].

## Therapeutic Perspective

Clinically, combating recurrent episodes of cortical spreading depolarizations, excitotoxicity, and neuroinflammation while supporting repair mechanisms is extremely important not only in acute ischemic stroke but also in other neurological diseases, such as subarachnoid hemorrhage, traumatic brain injury, and epilepsy or migraine. All of these diseases also lead to the degeneration of the NVU; hence, its protection as well as induction of its regeneration is very important.

Agonists of Y2R receptors might be promising protectants of the NVU against acute cerebral ischemia with reperfusion; however, many more animal studies are needed to confirm the proposed role of this peptide as a multitarget protectant. It would be particularly important to study the role of NPY 13–36 in the endogenous protection of the NVU.

A compound which protects neurovascular unit against ischemia/reperfusion could be a support of traditional recanalization methods but, although this idea seems attractive, there is a long way to go before clinical trials may be proposed.

The utility of NPY ligands as potential therapeutics is also limited due to the presence of the BBB; however, trials are currently being conducted on the delivery of peptides to the brain. It has been demonstrated in a few studies that when using cell-penetrating peptides (CPPs) as carriers of nonpenetrating compounds, the delivery of such compounds to cells is possible. In a study by Bright et al., an antagonist of δ-PKC (that did not penetrate the BBB) conjugated to a CPP significantly reduced the brain infarct area and neurological deficit after intra-arterial or intraperitoneal administration in rats after MCAO/R [138]. Moreover, several therapeutics conjugated to CPPs, after successful preclinical studies, have been tested in clinical trials [139].

## Summary of the Hypothetical Mechanisms of the Protection of NVU Against Acute Ischemia/Reperfusion by Activation of NPY2 Receptors

In this minireview, we summarize the basic knowledge of the putative mechanisms that underlie the defense against ischemia/reperfusion injury of the brain elicited by stimulation of Y2R. Currently, there is not much data about the exact mechanisms of Y2R in stroke, which is why most of the mechanisms proposed here come from research on diseases that have a partially similar pathophysiology to ischemic stroke. However, based on the presented experimental evidence, the mechanisms underlying the complex protective effects of Y2R agonists in ischemic stroke are worth to be studied.

**Fig. 3:**
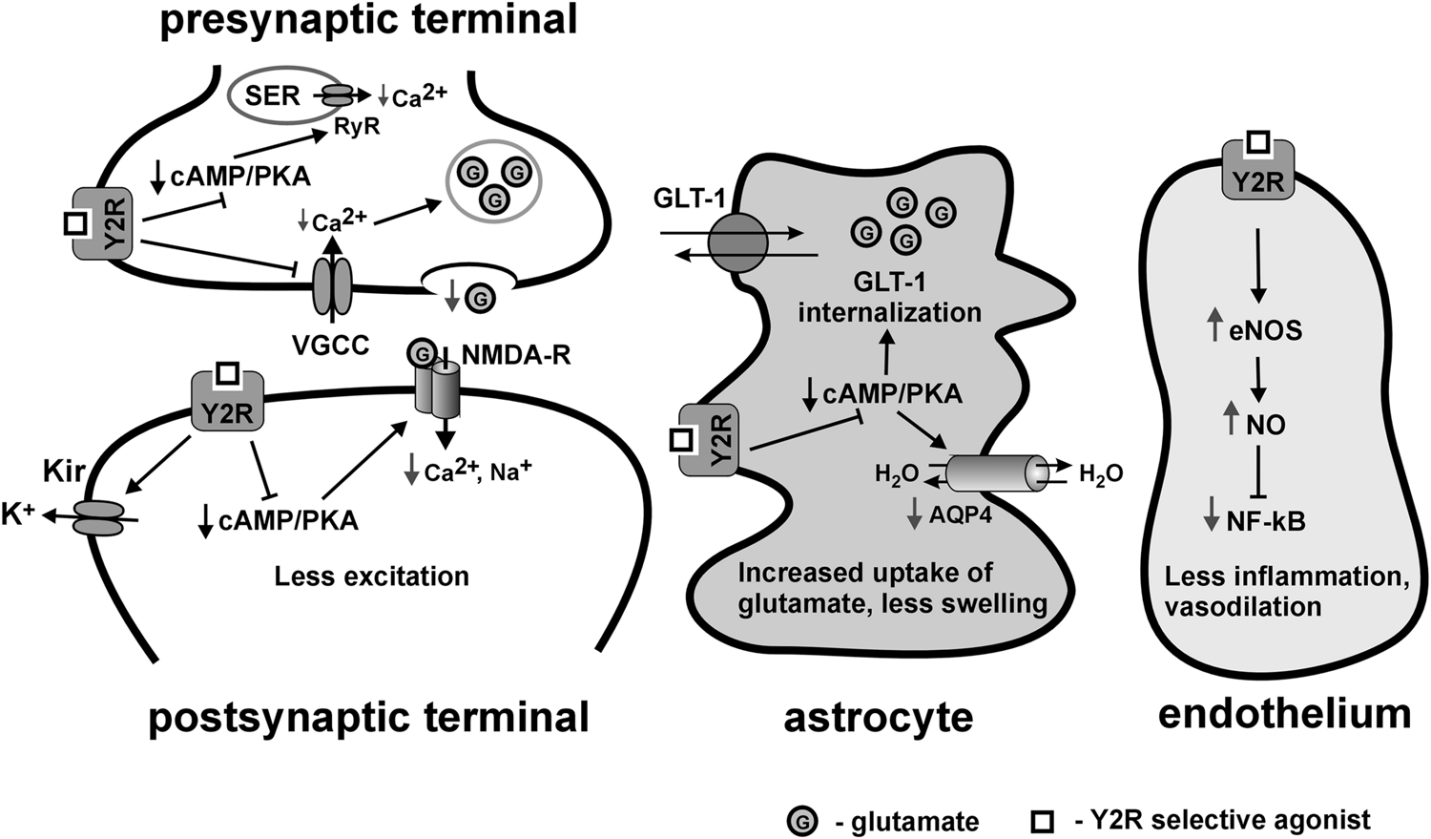
Hypothetical mechanisms of NVU protection by the activation of neuropeptide Y Y2 receptors (Y2R) in the NVU in the ischemic penumbra activated during acute phase of cerebral ischemia and reperfusion. The detailed description is provided in the text. Legend: AQP4 – aquaporin-4, Ca^2+^ – calcium ions, cAMP *– 3',5'-*cyclic adenosine monophosphate, eNOS – endothelial nitric oxide synthase, Kir – G protein-coupled inwardly rectifying potassium channel, GLT-1 – glutamate transporter 1, H_2_O – water molecule, K^+^ – potassium ion, Na^+^ – sodium ion, NF-kB – nuclear factor kappa B, NMDA-R – N-methyl-D*-*aspartate receptor (glutamate receptor), NO – nitric oxide, PKA – cAMP-dependent protein kinase (protein kinase A), SER – smooth endoplasmic reticulum*,* VGCC – voltage-gated calcium channel

The hypothetical mechanism of NVU protection by the activation of neuropeptide Y Y2 receptors (Y2R) in the ischemic penumbra during acute cerebral ischemia and early reperfusion is presented in Fig. 3. In the presynaptic site (upper left corner), Y2R stimulation leads to inhibition of the conductance of voltage-gated calcium channels (VGCC), which results in a decrease of the influx of extracellular calcium ions (Ca^2+^) into the presynaptic terminal. Simultaneously, reduction of adenylate cyclase and cAMP/PKA activity leads to decrease of ryanodine receptor (RyR) phosphorylation which reduces Ca^2+^ mobilization from the smooth endoplasmic reticulum (SER). The total concentration of Ca^2+^ in the presynaptic nerve ending does not rise as much as without Y2R stimulation, and less glutamate is released into the synaptic cleft. Activation of Y2R in the postsynaptic terminal (lower left corner) results in the decrease of intracellular cAMP/PKA activity and ultimately in the reduction of the excitability of the NMDA receptor. Furthermore, activation of Y2R results in opening of the G protein–coupled inwardly rectifying potassium channel (Kir), which allows K + ions to live the cell and hyperpolarizes the postsynaptic site. All in all, the transmission of the excitatory signal is decreased. In turn, activation of Y2R in the peri-synaptic astrocyte (middle) and decrease of intracellular cAMP/PKA activity results in the decreased internalization of membrane glutamate transporters (GLT-1) and allows more effective removal of the neurotransmitter from the synaptic cleft. All of these processes tend to quench neurons and may alleviate glutamate excitotoxicity and peri-infarct depolarizations. Moreover, inhibition of cAMP/PKA signaling in astrocytes by Y2R agonist decreases phosphorylation of AQP4 protein which results in the internalization of this water channel and reduces astrocytic edema in acute stroke. Simultaneously, activation of Y2R in the endothelial cells (right) stimulates nitric oxide synthase (eNOS) and nitric oxide (NO) production. NO ensures relaxation of smooth muscle cells of precapillary vessels, thus increasing blood flow to the penumbral/oligemic regions. Increased production of endothelial NO results also in the suppression of inflammation due to the inhibition of nuclear factor kappa B (NF-kB). Moreover, binding of NPY13-36 to Y2R on monocytes results in decrease of their activation and adhesion to the vascular wall (not shown). Inhibition of monocyte activation results in alleviation of oxidative/nitrosative stress, which may reduce the no-reflow effect and the production of ROS during reperfusion.

## References

[1] Kim J, Thayabaranathan T, Donnan GA, Howard G, Howard VJ, Rothwell P, et al. Global Stroke Statistics 2019. Int J Stroke. 2020;15(8):819–838. 10.1177/1747493020909545 32146867

[2] European Stroke Initiative Executive Committee; EUSI Writing Committee, Olsen TS, Langhorne P, Diener HC, Hennerici M, Ferro J, Sivenius J, et al. Cerebrovasc Dis. 2003;16:311–37. 10.1159/000072554 14584488

[3] Taschner CA, Treier M, Schumacher M, Berlis A, Weber J, Niesen W (2011). Mechanical thrombectomy with the penumbra recanalization device in acute ischemic stroke. J Neuroradiol.

[4] Samaniego EA, Linfante I, Dabus G (2012). Intra-arterial thrombolysis: tissue plasminogen activator and other thrombolytic agents. Tech Vasc Interv Radiol.

[5] Sussman ES, Connolly ES (2013). Hemorrhagic transformation: a review of the rate of hemorrhage in the major clinical trials of acute ischemic stroke. Front Neurol.

[6] Suzuki Y, Nagai N, Umemura K (2016). A review of the mechanisms of blood-brain barrier permeability by tissue-type plasminogen activatortreatment for cerebral ischemia. Front Cell Neurosci.

[7] Cohen DL, Kearney R, Griffiths M, Nadesalingam V, Bathula R (2015). Around 9% of patients with ischaemic stroke are suitable for thrombectomy. BMJ..

[8] Kuklina EV, Tong X, George MG, Bansil P (2012). Epidemiology and prevention of stroke: a worldwide perspective. Expert Rev Neurother.

[9] Del Zoppo GJ (2009). Inflammation and the neurovascular unit in the setting of focal cerebral ischemia. Neuroscience.

[10] Iadecola C, Anrather J (2012). The immunology of stroke: from mechanism to translation. Nat Med.

[11] Stroke Therapy Academic Industry Roundtable (STAIR) (1999). Recommendations for standards regarding preclinical neuroprotective and restorative drug development (STAIR). Stroke.

[12] Dirnagl U, Simon RP, Hallenbeck JM (2003). Ischemic tolerance and endogenous neuroprotection. Trends Neurosci.

[13] Hökfelt T, Bartfai T, Bloom F (2003). Neuropeptides: opportunities for drug discovery. Lancet Neurol.

[14] Eaton K, Sallee FR, Sah R (2007). Relevance of neuropeptide Y (NPY) in psychiatry. Curr Top Med Chem.

[15] Holzer P, Reichmann F, Farzi A, Neuropeptide Y (2012). peptide YY and pancreatic polypeptide in the gut-brain axis. Neuropeptides.

[16] Michel MC, Beck-Sickinger A, Cox H, Doods HN, Herzog H, Larhammar D (1998). XVI. International union of pharmacology recommendations for the nomenclature of neuropeptide Y, peptide YY, and pancreatic polypeptide receptors. Pharmacol Rev.

[17] Domin H, Przykaza Ł, Jantas D, Kozniewska E, Boguszewski PM, Śmiałowska M (2017). Neuropeptide Y Y2 and Y5 receptors as promising targets for neuroprotection in primary neurons exposed to oxygen-glucose deprivation and in transient focal cerebral ischemia in rats. Neuroscience.

[18] Przykaza L, Domin H, Boguszewski PM, Smialowska M, Kozniewska E (2016). Attenuation of postischemic functional deficits in rats with essential hypertension treated with NPY2R agonist. J Cereb Blood Flow Metab.

[19] Iadecola C (2017). The neurovascular unit coming of age: a journey through neurovascular coupling in health and disease. Neuron.

[20] Badaut J, Ashwal S, Obenaus A (2011). Aquaporins in cerebrovascular disease: a target for treatment of brain edema. Cerebrovasc Dis.

[21] Dirnagl U, Iadecola C, Moskowitz MA (1999). Pathobiology of ischaemic stroke: an integrated view. Trends Neurosci.

[22] Choi DW (1996). Ischemia-induced neuronal death. Trends Neurosci.

[23] Leist M, Nicotera P (1998). Apoptosis, excitotoxicity, and neuropathology. Exp Cell Res.

[24] Mutch WA, Hansen AJ (1984). Extracellular pH changes during spreading depression and cerebral ischemia: mechanisms of brain pH regulation. J Cereb Blood Flow Metab.

[25] Menyhárt Á, Zölei-Szénási D, Puskás T, Makra P, Orsolya MT, Szepes BÉ (2017). Spreading depolarization remarkably exacerbates ischemia-induced tissue acidosis in the young and aged rat brain. Sci Rep.

[26] Xiong ZG, Zhu XM, Chu XP, Minami M, Hey J, Wei WL (2004). Neuroprotection in ischemia: blocking calcium-permeable acid-sensing ion channels. Cell.

[27] O’Bryant Z, Vann KT, Xiong ZG (2014). Translational strategies for neuroprotection in ischemic stroke- focusing on acid-sensing ion channel 1a. Trans Stroke Res.

[28] Blaustein MP, Lederer WI (1999). Sodium/calcium exchange: its physiological implications. Physiol Rev.

[29] Cai X, Lytton J (2004). The cation/Ca(2+) exchange superfamily: phylogenetic analysis and structural implications. Mol Biol Evol.

[30] Jeffs GJ, Meloni BP, Bakker AJ, Knuckey NW (2007). The role of the Na(+)/Ca(2+) exchanger (NCX) in neurons following ischaemia. J Clin Neurosci.

[31] Tortiglione A, Picconi B, Barone I, Centonze D, Rossi S, Costa C (2007). Na^+^/Ca^2+^ exchanger maintains ionic homeostasis in the peri-infarct area. Stroke.

[32] Cuomo O, Gala R, Pignataro G, Boscia F, Secondo A, Scorziello A (2008). A critical role for the potassium-dependent sodium–calcium exchanger NCKX2 in protection against focal ischemic brain damage. J Neurosci.

[33] Matute C, Domercq M, Sánchez-Gómez MV (2006). Glutamate-mediated glial injury: mechanisms and clinical importance. Glia.

[34] Koźniewska E, Lisdat F, Ge B, Reszka R, Fukuuchi Y (2001). Impact of focal cerebral ischemia /reperfusion on the regulation of microcirculation in the cerebral cortex in rats. Tomita M.

[35] del Zoppo GJ, Mabuchi T (2003). Cerebral microvessel responses to focal ischemia. J Cereb Blood Flow Metab.

[36] Palomares SM, Cipolla MJ (2011). Vascular protection following cerebral ischemia and reperfusion. J Neurol Neurophysiol.

[37] Lauritzen M, Dreier JP, Fabricius M, Hartings JA, Graf R, Strong AJ (2011). Clinical relevance of cortical spreading depression in neurological disorders: migraine, malignant stroke, subarachnoid and intracranial hemorrhage, and traumatic brain injury. J Cereb Blood Flow Metab.

[38] Pietrobon D, Moskowitz MA (2014). Chaos and commotion in the wake of cortical spreading depression and spreading depolarizations. Nat Rev Neurosci.

[39] Gursoy-Ozdemir Y, Qiu J, Matsuoka N, Bolay H, Bermpohl D, Jin H (2004). Cortical spreading depression activates and upregulates MMP-9. J Clin Invest.

[40] Chang JC, Shook LL, Biag J, Nguyen EN, Toga AW, Charles AC (2010). Biphasic direct current shift, haemoglobin desaturation and neurovascular uncoupling in cortical spreading depression. Brain.

[41] Menyhárt Á, Farkas AE, Varga DP, Frank R, Toth R, Balint A (2018). Large-conductance Ca^2+^-activated potassium channels are potently involved in the inverse neurovascular response to spreading depolarization. Neurobiol Dis.

[42] del Zoppo GJ (2010). The neurovascular unit in the setting of stroke. J Int Med.

[43] Aronowski J, Strong R, Grotta JC (1997). Reperfusion injury: demonstration of brain damage produced by reperfusion after transient focal ischemia in rats. J Cereb Blood Flow Metab.

[44] Kuroda S, Siesjö BK (1997). Reperfusion damage following cerebral ischemia: pathophysiology and therapeutic windows. Clin Neurosci.

[45] Granger DN, Kvietys PR (2017). Reperfusion therapy-what’s with the obstructed, leaky and broken capillaries?. Pathophysiology.

[46] Bastide M, Bordet R, Pu Q, Robin E, Puisieux F, Dupuis B (1999). Relationship between inward rectifier potassium current impairment and brain injury after cerebral ischemia/reperfusion. J Cereb Blood Flow Metab.

[47] Pétrault O, Bastide M, Cotelle N, Gelé P, Gautier S, Laprais M (2004). The neuroprotectiveeffect of the antioxidantflavonoid derivate di-tert-butylhydroxyphenyl is parallel to the preventiveeffect on post-ischemicKir2.ximpairment but not to post-ischemicendothelialdysfunction. Naunyn Schmiedebergs Arch Pharmacol.

[48] Seitz I, Dirnagl U, Lindauer U (2004). Impaired vascular reactivity of isolated rat middle cerebral artery after cortical spreading depression in vivo. J Cereb Blood Flow Metab.

[49] Jourquin J, Tremblay E, Decanis N, Charton G, Hanessian S, Chollet AM (2003). Neuronal activity-dependent increase of net matrix metalloproteinase activity is associated with MMP-9 neurotoxicity after kainite. Eur J Neurosci.

[50] Pan W, Kastin AJ (2008). Tumor necrosis factor and stroke: role of the blood-brain barrier. Progress Neurobiol.

[51] Ritter LS, Orozco JA, Coull BM, McDonagh PF, Rosenblum WI (2000). Leukocyte accumulation and hemodynamic changes in the cerebral microcirculation during early reperfusion after stroke. Stroke.

[52] Zeller JA, Lenz A, Eschenfelder CC, Zunker P, Deuschl G (2005). Platelet-leukocyte interaction and platelet activation in acute stroke with and without preceding infection. Arterioscler Thromb Vasc Biol.

[53] Okada Y, Copeland BR, Mori E, Tung MM, Thomas WS, del Zoppo GJ (1994). P-selection and intercellular adhesion molecule-1 expression after focal cerebral ischemia and reperfusion. Stroke.

[54] Ten VS, Pinsky DJ (2002). Endothelial response to hypoxia: physiologic adaptation and pathologic dysfunction. Curr Opin Crit Care.

[55] Miller AA, Dusting GJ, Roulston CL, Sobey CG (2006). NADPH-oxidase activity is elevated inpenumbral and non-ischemic cerebral arteries following stroke. Brain Res.

[56] Radermacher KA, Wingler K, Langhauser F, Altenhöfer S, Kleikers P, Hermans JJ (2013). Neuroprotection after stroke by targeting NOX4 as a source of oxidative stress. Antioxid Redox Signal.

[57] Beckman JS, Koppenol WH (1996). Nitric oxide, superoxide, and peroxynitrite: the good, the bad and ugly. Am J Physiol.

[58] Iadecola C (1997). Bright and dark sides of nitric oxide in ischemic brain injury. Trends Neurosci.

[59] Beckman JS, Ye YZ, Chen J, Conger K (1996). The interactions of nitric oxide with oxygen radicals and scavengers in cerebral ischemic injury. Adv Neurol.

[60] Abe K, Tilan JU, Zukowska Z (2007). NPY and NPY receptors in vascular remodeling. Curr Top Med Chem.

[61] Robich MP, Matyal R, Chu LM, Feng J, Xu S-H, Laham RJ (2010). Effects of NPY on collateral development in a swine model of chronic myocardial ischemia. J Mol Cell Cardiol.

[62] Labelle M, St-Pierre S, Savard R, Boulanger Y (1997). Solution structure of neuropeptide tyrosine 13–36, a Y2 receptor agonist, as determined by NMR. Eur J Biochem.

[63] Mittapalli GK, Roberts E (2014). Ligands of the neuropeptide Y Y2 receptor. Bioorg Med Chem Lett.

[64] Ammar DA, Eadie DM, Wong DJ, Ma Y, Kolakowski LF, Yang-Feng TL (1996). Characterization of the human type 2 neuropeptide Y receptor gene (NPY2R) and localization to the chromosome 4q region containing the type 1 neuropeptide Y receptor gene. Genomics.

[65] Nakamura M, Aoki Y, Hirano D (1996). Cloning and functional expression of a cDNA encoding a mouse type 2 neuropeptide Y receptor. Biochim Biophys Acta.

[66] Lutz CM, Frankel WN, Richards J, Thompson DA (1997). Neuropeptide Y receptor genes on human chromosome 4q31-q32 map to conserved linkage groups on mouse chromosomes 3 and 8. Genomics.

[67] Goumain M, Voisin T, Lorinet AM, Ducroc R, Tsocas A, Rozé C (2001). The peptide YY-preferring receptor mediating inhibition of small intestinal secretion is a peripheral Y(2) receptor: pharmacological evidence and molecular cloning. Mol Pharmacol.

[68] Larhammar D (1996). Structural diversity of receptors for neuropeptide Y, peptide YY and pancreatic polypeptide. Regul Pept.

[69] Caberlotto L, Fuxe K, Rimland JM, Sedvall G, Hurd YL (1998). Regional distribution of neuropeptide Y Y2 receptor messenger RNA in the human post mortem brain. Neuroscience.

[70] Parker RM, Herzog H (1999). Regional distribution of Y-receptor subtype mRNAs in rat brain. Eur J Neurosci.

[71] Shaw JL, Gackenheimer SL, Gehlert DR (2003). Functional autoradiography of neuropeptide Y Y1 and Y2 receptor subtypes in rat brain using agonist stimulated [35S]GTPgammaSbinding. J Chem Neuroanat.

[72] Ghamari-Langroudi M, Colmers WF, Cone RD (2005). PYY3–36 inhibits the action potential firing activity of POMC neurons of arcuate nucleus through postsynaptic Y2 receptors. Cell Metab.

[73] Stanić D, Mulder J, Watanabe M, Hökfelt T (2011). Characterization of NPY Y2 receptor protein expression in the mouse brain. II. Coexistence with NPY, the Y1 receptor, and other neurotransmitter-related molecules. J Comp Neurol.

[74] Mantyh PW, Allen CJ, Rogers S, DeMaster E, Ghilardi JR, Mosconi T (1994). Some sensory neurons express neuropeptide Y receptors: potential paracrine inhibition of primary afferent nociceptors following peripheral nerve injury. J Neurosci.

[75] Abounader R, Elhusseiny A, Cohen Z, Olivier A, Stanimirovic D, Quirion R (1999). Expression of neuropeptide Y receptors mRNA and protein in human brain vessels and cerebromicrovascular cells in culture. J Cereb Blood Flow Metab.

[76] You J, Edvinsson L, Bryan RM (2001). Neuropeptide Y-mediated constriction and dilation in rat middle cerebral arteries. J Cereb Blood Flow Metab.

[77] Hökfelt T, Broberger C, Zhang X, Diez M, Kopp J, Xu Z (1998). Neuropeptide Y: some viewpoints on a multifaceted peptide in the normal and diseased nervous system. Brain Res Rev.

[78] Reichmann F, Holzer P (2016). Neuropeptide Y: a stressful review. Neuropeptides.

[79] Brothers SP, Wahlestedt C (2010). Therapeutic potential of neuropeptide Y (NPY) receptor ligands. EMBO Mol Med.

[80] Tasan RO, Verma D, Wood J, Lach G, Hörmer B, de Lima TC (2016). The role of Neuropeptide Y in fear conditioning and extinction. Neuropeptides.

[81] Smialowska M, Domin H, Zieba B, Koźniewska E, Michalik R, Piotrowski P (2009). Neuroprotective effects of neuropeptide Y-Y2 and Y5 receptor agonists in vitro and in vivo. Neuropeptides.

[82] Allen GV, Cheung RT, Cechetto DF (1995). Neurochemical changes following occlusion of the middle cerebral artery in rats. Neuroscience.

[83] Higgins JPT, Thompson SG, Deeks JJ, Altman DG (2003). Measuring inconsistency in meta-analyses. BMJ.

[84] Higgins JPT, Li T, Deeks JJ. Choosing effect measures and computing estimates of effect. In: Higgins JPT, Thomas J, Chandler J, Cumpston M, Li T, J. Page MJ, Welch VA, editors. Cochrane handbook for systematic reviews of interventions. The Cochrane Collaboration and John Wiley & Sons Ltd.; 2019. pp. 149–50.

[85] Goh JX, Hall JA, Rosenthal R (2016). Mini meta-analysis of your own studies: some arguments on why and a primer on how. Soc Pers Psyc Comp.

[86] Fukuyama N, Takizawa S, Ishida H, Hoshiai K, Shinohara Y, Nakazawa H (1998). Peroxynitrite formation in focal cerebral ischemia-reperfusion in rats occurs predominantly in the peri-infarct region. J Cereb Blood Flow Metab.

[87] Fabian RH, DeWitt DS, Kent TA (1995). In vivo detection of superoxide anion production by the brain using a cytochrome c electrode. J Cereb Blood Flow Metab.

[88] Yagita Y, Kitagawa K, Oyama N, Yukami T, Watanabe A, Sasaki T (2013). Functional deterioration of endothelial nitric oxide synthase after focal cerebral ischemia. J Cereb Blood Flow Metab.

[89] Lefer AM (1997). Nitric oxide Nature’s naturally occurring leukocyte inhibitor. Circulation.

[90] Cooper DM, Mons N, Karpen JW (1995). Adenylyl cyclases and the interaction between calcium and cAMP signaling. Nature.

[91] Bergantin LB, Jurkiewicz A, Garcia AG, Caricati-Neto A (2015). A calcium paradox in the context of neurotransmission. J Pharm Pharmacol.

[92] Chavez-Noriega LE, Stevens CF (1994). Increased transmitter release at excitatory synapses produced by direct activation of adenylate cyclase in rat hippocampal slices. J Neurosci.

[93] Westphal RS, Tavalin SJ, Lin JW, Alto NM, Fraser ID, Langeberg LK (1999). Regulation of NMDA receptors by an associated phosphatase-kinase signaling complex. Science.

[94] Threlfell S, West AR (2013). Review: Modulation of striatal neuron activity by cyclic nucleotide signaling and phosphodiesterase inhibition. Basal Ganglia.

[95] Colwell CS, Levine MS (1995). Excitatory synaptic transmission in neostriatalneurons: regulation by cyclic AMP-dependent mechanisms. J Neurosci.

[96] Threlfell S, Sammut S, Menniti FS, Schmidt CJ, West AR (2009). Inhibition of phosphodiesterase 10A increases the responsiveness of striatal projection neurons to cortical stimulation. J Pharmacol Exp Ther.

[97] Domańska-Janik K, Pylova S (1992). Postreceptor modulation of cAMP accumulation in rat brain particulate fraction after ischemia – involvement of protein kinase C. Mol Chem Neuropathol.

[98] Wang Q, Zhou FM (2019). cAMP-producing chemogenetic and adenosine A2a receptor activation inhibits the inwardly rectifying potassium current in striatal projection neurons. Neuropharmacology.

[99] Krivánek J (1976). Adenosine 3’,5’-monophosphate in rat cerebral cortex: effect of potassium ions in vivo (cortical spreading depression). J Neurochem.

[100] Krivánek J (1983). Vanadate and brain adenylate cyclase. Effect of spreading depression. Neuroscience.

[101] Tupal S, Faingold C (2010). Inhibition of adenylyl cyclase in amygdala blocks the effect of audiogenic seizure kindling in genetically epilepsy-prone rats. Neuropharmacology.

[102] Nishizaki T, Nagai K, Nomura T, Tada H, Kanno T, Tozaki H (2002). A new neuromodulatory pathway with a glial contribution mediated via A2a adenosine receptors. Glia.

[103] Guillet BA, Velly LJ, Canolle B, Masmejean FM, Nieoullon AL, Pisano P (2005). Differential regulation by protein kinases of activity and cell surface expression of glutamate transporters in neuron-enriched cultures. Neurochem Int.

[104] Adolph O, Köster S, Räth M, Georgieff M, Weigt HU, Engele J (2007). Rapid increase of glial glutamate uptake via blockade of the protein kinase A pathway. Glia.

[105] Song Y, Gunnarson E (2012). Potassium dependent regulation of astrocyte water permeability is mediated by cAMP signaling. PLoS One..

[106] Kitchen P, Salman MM, Halsey AM, Clarke-Bland C, MacDonald JA, Ishida H (2020). Targeting Aquaporin-4 subcellular localizationto treat central nervous system edema. Cell.

[107] Yao X, Smith AJ, Jin BJ, Zador Z, Manley GT, Verkman AS (2014). Aquaporin-4 regulates the velocity and frequency of cortical spreading depression in mice. Glia.

[108] Vezzani A, Sperk G (2004). Overexpression of NPY and Y2 receptors in epileptic brain tissue: an endogenous neuroprotective mechanism in temporal lobe epilepsy?. Neuropeptides.

[109] Woldbye DP, Angehagen M, Gøtzsche CR, Elbrønd-Bek H, Sørensen AT, Christiansen SH (2010). Adeno-associated viral vector-induced overexpression of neuropeptide Y Y2 receptors in the hippocampus suppresses seizures. Brain.

[110] Xu X, Guo F, He Q, Cai X, Min D, Wang Q (2014). Altered expression of neuropeptide Y, Y1 and Y2 receptors, but not Y5 receptor, within hippocampus and temporal lobe cortex of tremor rats. Neuropeptides.

[111] Ledri M, Sørensen AT, Madsen MG, Christiansen SH, Ledri LN, Cifra A (2015). Differential effect of neuropeptides on excitatory synaptic transmission in human epileptic hippocampus. J Neurosci.

[112] Wickham J, Ledri M, Bengzon J, Jespersen B, Pinborg LH, Englund E (2019). Inhibition of epileptiform activity by neuropeptide Y in brain tissue from drug-resistant temporal lobe epilepsy patients. Sci Rep.

[113] Acuna-Goycolea C, Tamamaki N, Yanagawa Y, Obata K, van den Pol AN (2005). Mechanisms of neuropeptide Y, peptide YY, and pancreatic polypeptide inhibition of identified green fluorescent protein-expressing GABA neurons in the hypothalamic neuroendocrine arcuate nucleus. J Neurosci.

[114] Liu YQ, Qiu F, Qiu CY, Cai Q, Zou P, Wu H (2012). Cannabinoids inhibit acid-sensing ion channel currents in rat dorsal root ganglion neurons. PLoS One.

[115] Duan B, Wang YZ, Yang T, Chu XP, Yu Y, Huang Y (2011). Extracellular spermine exacerbates ischemic neuronal injury through sensitization of ASIC1a channels to extracellular acidosis. J Neurosci.

[116] dos Santos VV, Santos DB, Lach G, Rodrigues AL, Farina M, De Lima TC (2013). Neuropeptide Y (NPY) prevents depressive-like behavior, spatial memory deficits and oxidative stress following amyloid-β administration in mice. Behav Brain Res.

[117] Chen GY, Nunez G (2010). Sterile inflammation: sensing and reacting to damage. Nat Rev Immunol.

[118] Woods TA, Du M, Carmody A, Peterson KE (2015). Neuropeptide Y negatively influences monocyte recruitment to the central nervous system during retrovirus infection. J Virol.

[119] Fatoba O, Kloster E, Reick C, Saft C, Gold R, Epplen JT (2018). Activation of NPY-Y2 receptors ameliorates disease pathology in the R6/2 mouse and PC12 cell models of Huntington’s disease. Exp Neurol.

[120] Gonçalves J, Ribeiro CF, Malva JO, Silva AP (2012). Protective role of neuropeptide Y Y_2_ receptors in cell death and microglial response following methamphetamine injury. Eur J Neurosci.

[121] Hayashi T, Noshita N, Sugawara T, Chan PH (2003). Temporal profile of angiogenesis and expression of related genes in the brain after ischemia. J Cereb Blood Flow Metab.

[122] Sun Y, Jin K, Xie L, Childs J, Mao XO, Logvinova A (2003). VEGF-induced neuroprotection, neurogenesis, and angiogenesis after focal cerebral ischemia. J Clin Invest.

[123] Krupiński J, Kałuza J, Kumar P, Kumar S, Wang JM (1993). Some remarks on the growth-rate and angiogenesis of microvessels in ischemic stroke. Morphometric and immunocytochemical studies. Patol Pol.

[124] Ergul A, Alhusban A, Fagan SC (2012). Angiogenesis: a harmonized target for recovery after stroke. Stroke..

[125] Kanazawa M, Takahashi T, Ishikawa M, Onodera O, Shimohata T, Del Zoppo GJ (2019). Angiogenesis in the ischemic core: a potential treatment target?. J Cereb Blood Flow Metab.

[126] Lee EW, Michalkiewicz M, Kitlinska J, Kalezic I, Switalska H, Yoo P (2003). Neuropeptide Y induces ischemic angiogenesis and restores function of ischemic skeletal muscles. J Clin Invest.

[127] Stanic D, Paratcha G, Ledda F, Herzog H, Kopin AS, Hökfelt T (2008). Peptidergic influences on proliferation, migration, and placement of neural progenitors in the adult mouse forebrain. PNAS.

[128] Saraf R, Mahmood F, Amir R, Matyal R (2016). Neuropeptide Y is an angiogenic factor in cardiovascular regeneration. Eur J Pharmacol.

[129] Zukowska-Grojec Z, Karwatowska-Prokopczuk E, Rose W, Rone J, Movafagh S, Ji H (1998). Neuropeptide Y: a novel angiogenic factor from the sympathetic nerves and endothelium. Circ Res.

[130] Ghersi G, Chen W, Lee EW, Żukowska Z (2001). Critical role of dipeptidyl peptidase IV in neuropeptide Y-mediated endothelial cell migration in response to wounding. Peptides.

[131] Conn G, Bayne ML, Soderman DD, Kwok PW, Sullivan KA, Palisi TM (1990). Amino acid and cDNA sequences of a vascular endothelial cell mitogen that is homologous to platelet-derived growth factor. Proc Natl Acad Sci USA.

[132] Kitlińska J, Lee EW, Movafagh S, Pons J, Zukowska Z (2002). Neuropeptide Y-induced angiogenesis in aging. Peptides.

[133] Movafagh S, Hobson JP, Spiegel S, Kleinman HK, Zukowska Z (2006). Neuropeptide Y induces migration, proliferation, and tube formation of endotheliual cells bimodally via Y1, Y2, and Y5 receptors. FASEB J.

[134] Geloso MC, Corvino V, Di Maria V, Marchese E, Michetti F (2015). Cellular targets for neuropeptide Y-mediated control of adult neurogenesis. Front Cell Neurosci.

[135] Alvaro AR, Martins J, Araújo IM, Rosmaninho-Salgado J, Ambrósio AF, Cavadas C (2008). Neuropeptide Y stimulates retinal neural cell proliferation–involvement of nitric oxide. J Neurochem.

[136] Silva AP, Carvalho AP, Carvalho CM, Malva JO (2003). Functional interaction between neuropeptide Y receptors and modulation of calcium channels in the rat hippocampus. Neuropharmacology.

[137] Teng H, Zhang ZG, Wang L, Zhang RL, Zhang L, Morris D (2008). Coupling of angiogenesis and neurogenesis in cultured endothelial cells and neural progenitor cells after stroke. J Cereb Blood Flow Metab.

[138] Bright R, Raval AP, Dembner JM, Pérez-Pinzón MA, Steinberg GK, Yenari MA (2004). Protein kinase C delta mediates cerebralreperfusion injury in vivo. J Neurosci.

[139] Guidotti G, Brambilla L, Rossi D (2017). Cell-penetrating peptides: from basic research to clinic. Trends Pharmacol Sci.

